# Enhanced Sensitivity Mach–Zehnder Interferometer-Based Tapered-in-Tapered Fiber-Optic Biosensor for the Immunoassay of C-Reactive Protein

**DOI:** 10.3390/bios15020090

**Published:** 2025-02-06

**Authors:** Lei Xiao, Xinghong Chen, Xuejin Li, Jinghan Zhang, Yan Wang, Dongqing Li, Xueming Hong, Yonghong Shao, Yuzhi Chen

**Affiliations:** 1 College of Physics and Optoelectronic Engineering, Shenzhen University, Shenzhen 518060, China; 2050453015@email.szu.edu.cn (L.X.); 2250453009@email.szu.edu.cn (X.C.); lixuejin@szu.edu.cn (X.L.); zhangjinghan@mail.scuec.edu.cn (J.Z.); 2150453019@email.szu.edu.cn (Y.W.); 2210452034@email.szu.edu.cn (D.L.); xmhong@szu.edu.cn (X.H.); 2 Shenzhen Engineering Laboratory for Optical Fiber Sensors and Networks, Shenzhen 518060, China; 3 Shenzhen Tian’an Zhiyuan Sensor Technology Co., Ltd., Shenzhen 518060, China; 4 Shenzhen Key Laboratory of Sensor Technology, Shenzhen 518060, China; 5 School of Science, The Chinese University of Hong Kong, Shenzhen 518172, China

**Keywords:** fiber-optic biosensors, tapered-in-tapered fibers, C-reactive protein detection

## Abstract

A Mach–Zehnder interferometer-based tapered-in-tapered fiber-optic biosensor was introduced in this paper. By integrating a micro-tapered fiber into a single tapered fiber structure, the design enhances sensitivity, signal-to-noise ratio, and resolution capability, while reducing the length of the sensing fiber. Through simulation analysis, it was found that the tapered-in-tapered fiber significantly improved the refractive index detection sensitivity by exciting a stronger evanescent field effect. The experimental comparison between the tapered-in-tapered fiber and traditional tapered fiber showed a 1.7-fold increase in sensitivity, reaching 3266.78 nm/RIU within the refractive index range of 1.3326 to 1.3414. Furthermore, to expand its application prospects in the biomedical field, glutaraldehyde cross-linking technology was used to immobilize C-reactive protein (CRP) antibodies on the surface of the tapered-in-tapered fiber, successfully creating a biosensing platform for the specific recognition of CRP. The experimental results demonstrate that this novel biosensor can rapidly and accurately detect CRP molecules at different concentrations with a detection limit of 0.278 μg/mL, and that it exhibits good selectivity and repeatability. This tapered-in-tapered fiber-optic biosensor provides new insights into the development of high-performance fiber-optic immunosensors and shows broad application potential in immunology research and early disease diagnosis.

## 1. Introduction

C-reactive protein (CRP) is an acute-phase protein produced by the liver, forming part of the immune system’s response [1]. In healthy adults, CRP levels are typically low, generally below 1 mg/L [2]; however, under pathological conditions such as infections, trauma, inflammation, or tumors, its concentration significantly increases [3]. As an important inflammatory marker, the level of CRP can reflect the degree of inflammation in the body and the progression of certain diseases. Therefore, in clinical practice, CRP testing is widely used in various fields such as inflammatory diseases [4], cardiovascular diseases [5], and postoperative complications monitoring [6]. Particularly in cardiovascular disease diagnosis, high-sensitivity CRP is considered one of the key indicators for predicting the risk of cardiovascular events [7]. Given this, developing rapid, sensitive, and highly specific CRP detection technologies is crucial for early disease diagnosis and therapeutic monitoring.

Traditional methods for CRP detection mainly include enzyme-linked immunosorbent assay (ELISA) [8], turbidimetry [9], and fluorescent labeling [10]. Although these methods have high sensitivity and specificity, they often require complex operational steps, long detection times, and expensive instrumentation support, making it difficult to meet the needs of point-of-care testing (POCT) [11]. In recent years, with the development of biosensing technology, immunosensors have received widespread attention due to their simple and quick operation process, short response time, and high sensitivity, gradually becoming one of the important means of CRP detection [12]. Among various types of immunosensors, fiber-optic immunosensors have been intensively studied and applied in the biomedical field due to their miniaturized design, high sensitivity, and electromagnetic interference resistance [13]. These sensors use fiber optics as the medium for sensing and transmitting biological signals and capture target antigens by immobilizing specific antibodies on the fiber surface, thereby achieving real-time monitoring of target molecules. Based on different working principles, fiber-optic immunosensors can be categorized into several types, including those based on fiber grating [14], surface plasmon resonance (SPR) [15], lossy mode resonance [16], interferometer [17], etc. For example, in 2021, Esposito et al. reported a long-period grating fiber-optic sensor coated with graphene oxide to enable CRP detection [18]; in 2023, Cao et al. reported a fiber-optic SPR biosensor for the specific detection of CRP [19]; and in 2024, Cierpiak et al. reported an interferometry-based fiber-optic sensor for the assessment of CRP in human urine [20]. Tapered fibers are particularly favored by researchers due to their simple structure, ease of fabrication, and higher sensitivity [21]. Moreover, the larger surface area of tapered fibers provides more possibilities for subsequent biofunctionalization. However, the sensitivity enhancement of traditional tapered fibers is achieved by reducing the diameter of the sensing fiber while elongating it. This can bring inconvenience to detection and increase the amount of biological sample required.

In 2021, Wang et al. first proposed a localized surface plasmon resonance (LSPR) biosensor based on a tapered-in-tapered fiber structure [22]. They further tapered down a conventional taper to a diameter of 40 μm to generate more optical field excitation of plasmon waves. In 2022, Gong et al. built upon the traditional tapered structure and further tapered it down to 20 μm, introducing a mode interferometer based on the tapered-in-tapered fiber structure, thereby enhancing the detection performance of the sensor [23]. In the same year, Kumar et al. successfully excited the LSPR effect using the tapered-in-tapered fiber structure, achieving efficient detection of cresol [24]. Previous studies have demonstrated that the tapered-in-tapered fiber structure can more effectively excite the evanescent field, thereby improving the performance of the sensor.

This study aimed to explore a fiber-optic biosensor structure—the tapered-in-tapered fiber-optic biosensor. The design integrates a micro-tapered section into a single tapered fiber structure, enhancing the contrast of interference fringes, reducing the full width at half maximum (FWHM) of the interference fringes and significantly improving detection sensitivity while also shortening the length of the sensing fiber. The experimental results show that this novel sensor exhibited excellent performance in liquid refractive index (RI) tests, achieving an ultra-high sensitivity of 3266.78 nm/RIU when the solution RI ranged from 1.3326 to 1.3414, approximately 1.7 times higher than that of traditional tapered fiber-optic sensors. Furthermore, we successfully immobilized human CRP antibodies (Anti-h CRP) onto the surface of the tapered-in-tapered fiber using glutaraldehyde cross-linking technology, endowing the sensor with the ability to specifically recognize CRP. After validation, this fiber-optic biosensor can accurately determine CRP content within different concentration ranges with a detection limit (LOD) of 0.278 μg/mL, while maintaining good repeatability and stability. This research achievement not only opens a new pathway for the development of high-performance fiber-optic immunosensors but also provides strong technical support for the rapid and accurate detection of biomolecules.

## 2. Materials and Methods

### 2.1. Sensing Principle

**Figure 1:**
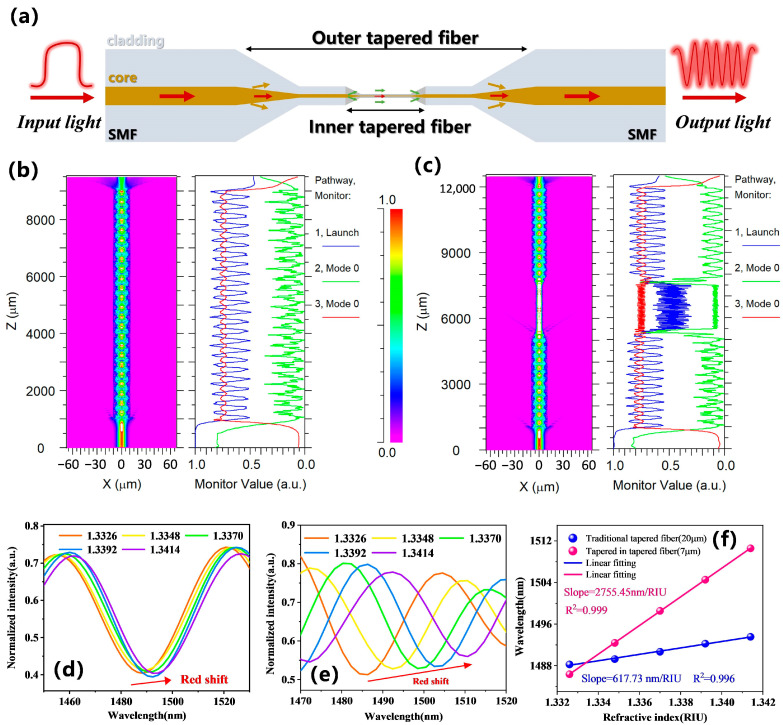
Comparative analysis between the tapered-in-tapered fiber-optic sensor and the traditional tapered fiber-optic sensor: (**a**) Schematic diagram of the tapered-in-tapered fiber sensing structure. (**b**) Longitudinal light field energy distribution of a traditional tapered fiber structure with a waist diameter of 20 µm. (**c**) Longitudinal light field energy distribution of a tapered-in-tapered fiber structure with a micro-tapered fiber waist diameter of 7 µm. (**d**) Simulated transmission spectra of the traditional tapered fiber sensing structure at different RIs. (**e**) Simulated transmission spectra of the tapered-in-tapered fiber sensing structure at different RIs. (**f**) Simulation comparison of the RI sensitivity for both sensing structures.

The proposed fiber-optic sensor consists of a two-stage tapered structure: the first stage is a tapered fiber, and the second stage is a micro-tapered fiber structure within the tapered fiber. Figure 1a shows a schematic diagram of the tapered-in-tapered fiber-optic sensor, where the first stage (outer tapered fiber) can be regarded as a Mach–Zehnder interferometer (MZI). As the diameter of the fiber decreases in the tapered transition region, light propagating in the fiber splits into two beams. One beam, which propagates in the waist area of the tapered fiber, serves as the reference light; while the other beam, which travels at the surface of the tapered fiber’s waist, acts as the measurement light. The two beams couple at the back end of the fiber’s transition region, generating an interference phenomenon. Within the waist area of the first stage structure, a second-stage inner tapered fiber is further nested. The integration of the inner tapered fiber allows for a further reduction in the waist diameter without extending the length of the sensing area too much, and it can excite a stronger evanescent field [25] to stimulate additional high-order cladding mode coupling that generates interference. Therefore, the output light intensity of the final tapered-in-tapered fiber-optic interferometer is given by [26]:

(1) $$I=I_{1}+\sum_{i=1}^{n}I_{2}^{i}+2\sum_{i=1}^{n}\sqrt{I_{1}I_{2}^{i}}\cos\Delta \varphi^{i}$$

where $I_{1}$ and $I_{2}^{i}$ are the intensities of the core mode and other high-order cladding modes, respectively, and ${\Delta \varphi }^{i}$ is the phase difference between them, which can be expressed as [27]

(2) $$\Delta \varphi^{i}=\frac{2\pi \left(\Delta n_{\mathrm{eff}}^{o}L_{1}+\Delta n_{\mathrm{eff}}^{i}L_{2}\right)}{\lambda }$$

where $L_{1}$ and $L_{2}$ are the sensing lengths of the outer and inner tapered fibers, respectively, and ${\Delta n}_{\mathrm{eff}}^{o}$ and ${\Delta n}_{\mathrm{eff}}^{i}$ are the effective RI differences of the outer and inner tapered fibers. λ is the input wavelength of the light source. As the RI of the external environment changes, ${\Delta n}_{\mathrm{eff}}^{o}$ and ${\Delta n}_{\mathrm{eff}}^{i}$ in the tapered-in-tapered fiber-optic structure will also change accordingly, leading to a shift in the transmission spectrum. The RI sensitivity (S) at the response wavelength ($\lambda_{\mathrm{res}}$) can be expressed as [28]

(3) $$S=\frac{d\lambda_{\mathrm{res}}}{dn_{\mathrm{SRI}}}=\frac{\lambda_{\mathrm{res}}\partial \left(\Delta n_{\mathrm{eff}}\right)/\partial n_{\mathrm{SRI}}}{\Delta n_{\mathrm{eff}}-\lambda_{\mathrm{res}}\partial \left(\Delta n_{\mathrm{eff}}\right)/\partial \lambda_{\mathrm{res}}}=\frac{\lambda_{\mathrm{res}}}{G}\cdot \frac{\partial \left(\Delta n_{\mathrm{eff}}\right)}{\partial n_{\mathrm{SRI}}}$$

where $n_{\mathrm{SRI}}$ is the RI of the external environment, and $\partial \left(\Delta n_{\mathrm{eff}}\right)/\partial n_{\mathrm{SRI}}$ represents the effect of changes in the external environment’s RI on ${\Delta n}_{\mathrm{eff}}$. G is the group effective RI difference between the core mode and high-order cladding modes. From the formula, it can be seen that the sensor’s response sensitivity is primarily determined by three factors: $\lambda_{\mathrm{res}}$, $\partial \left(\Delta n_{\mathrm{eff}}\right)/\partial n_{\mathrm{SRI}}$, and G.

### 2.2. Simulation Analysis

Before fabricating the tapered-in-tapered fiber, we conducted a simulation analysis of both traditional tapered fiber structures and tapered-in-tapered fiber structures using the beam propagation method in RSOFT software (version 2018.12). For the traditional tapered fiber structure, the taper transition region was set to 500 µm, with a uniform waist area length of 8000 µm and a diameter of 20 µm. The tapered-in-tapered fiber structure included an integrated micro-tapered fiber, with its transition region also set at 500 µm, a uniform micro-waist area length of 2000 µm, and a diameter of 7 µm. At an incident light wavelength of 1550 nm and under an RI detection condition of 1.3326, Figure 1b,c show the longitudinal light field distributions for the traditional tapered fiber and tapered-in-tapered fiber structures, respectively. The blue energy lines represent the total energy distribution across the fiber waist, while the green and red energy lines denote the energy distribution of modes propagating inside and on the surface of the fiber waist. From the normalized energy monitoring results, it can be seen that when light propagated to the fiber’s transition region, energy exchange occurred between core modes and cladding modes, with most of the core mode energy leaking into the cladding and propagating there. Ultimately, at the back end of the fiber’s transition region, coupling occurred between core and cladding modes. The light field transmission distribution of the tapered-in-tapered fiber showed that when light reached the transition region of the micro-tapered fiber, energy further leaked from the fiber, and within the 7 µm micro-waist area, due to the further reduction in diameter, light energy propagation was more confined, significantly increasing local light field intensity and concentrating the light field energy.

To further compare the RI response capabilities of the two types of tapered fibers, we used the wavelength scanning function of RSOFT software to calculate the detection transmission spectra of the traditional tapered fiber structure (with a waist diameter of 20 µm) and the tapered-in-tapered fiber structure (with a micro-waist diameter of 7 µm) over a wavelength range of 1450 nm to 1650 nm and a RI range of 1.3326 to 1.3414, as shown in Figure 1d,e. As the detected RI increased, a redshift phenomenon was observed in the transmission spectrum, consistent with theoretical analysis. Figure 1f displays the trend of response wavelength changes with RI and their linear fitting analysis results for both traditional tapered fiber and tapered-in-tapered fiber structures. It can be seen that the simulated RI sensitivity of the tapered-in-tapered fiber structure was 2755.45 nm/RIU, significantly higher than the 617.73 nm/RIU sensitivity of the traditional tapered fiber structure. This enhancement in sensitivity was due to the integration of the micro-tapered fiber, which further reduced the fiber waist diameter and excited stronger evanescent waves, thereby significantly improving the RI detection performance. To further enhance the rationality of the design, we conducted a simulation analysis on the response capability of different waist diameters of micro-tapered fiber sensors to external RI changes (see Supplementary Materials for the optimization analysis of the waist diameter of the micro-tapered fiber). The simulation results indicate that reducing the waist diameter of the fiber significantly improved RI sensitivity. Of course, further decreasing the waist diameter can enhance the detection sensitivity of the sensor even more. However, considering the difficulty of sensor fabrication, a waist diameter of 7 μm and a length of 2000 μm were chosen for further research.

### 2.3. Preparation of the Sensor

**Figure 2:**
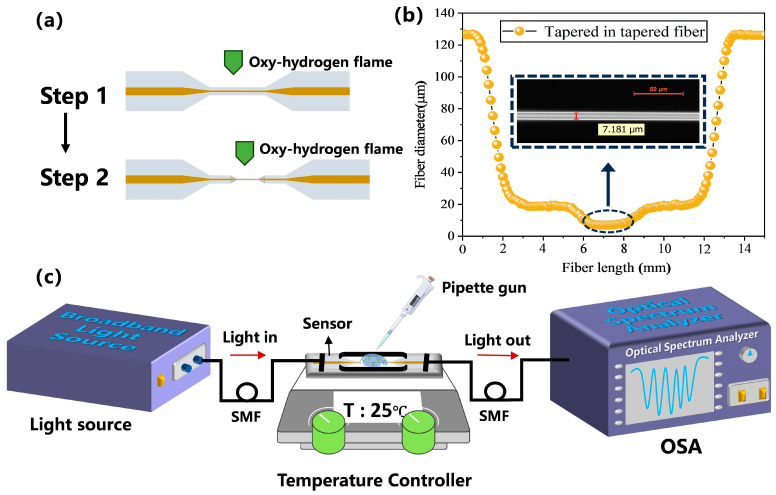
(**a**) Schematic diagram of the sensor fabrication process. (**b**) Dimensional scan data of the experimentally produced tapered-in-tapered fiber-optic sensor (inset: microscopic image of the micro-waist diameter). (**c**) Schematic diagram of the experimental detection setup.

Figure 2a illustrates the sensor manufacturing process. The preparation of the tapered-in-tapered fiber-optic sensor primarily involved two steps: Step 1: A commercial fiber-optic fusion splicer (model: AFBT-8000, Shandong Kaipule Optoelectronic Technology Co., Ltd., Tai’an, China) was utilized to taper the single-mode fiber. Based on the simulated analysis of the tapered fiber parameters, in this step, the length of the tapered region was set to 500 µm, the uniform waist area length was set to 8000 µm, and the waist diameter was set to 20 µm. This step produced a traditional tapered fiber-optic sensor. Building on Step 1, Step 2 was carried out: The flame was moved to the 20 µm diameter waist area of the tapered fiber to create a micro-tapered fiber. Based on the simulated analysis of the tapered-in-tapered fiber parameters, during this process, the length of the micro-tapered fiber’s gradual transition region was set to 500 µm, the uniform micro-waist area length was set to 2000 µm, and the micro-waist diameter was set to 7 µm. Finally, the completed tapered-in-tapered fiber-optic sensor was fixed onto the support frame of the experimental platform using UV-curable adhesive to ensure its stability and firmness. Figure 2b shows the structural dimension scan data of the experimentally produced tapered-in-tapered fiber-optic sensor, where the built-in tapered fiber micro waist diameter was approximately 7.18 µm. In the second tapering process, excessively high temperatures and prolonged heating times can cause bending or irreversible structural changes in the tapered fiber. Excessively high tapering speeds can lead to increased mechanical stress, which can further result in the fracture of the tapered fiber during the heating process. Therefore, in the tapered-in-tapered fiber manufacturing process, to minimize the potential impact of the second tapering process on the structural integrity and optical characteristics of the first tapered structure, the heating power, heating time, and stretching speed in the second tapering procedure were reduced. We experimentally prepared five tapered-in-tapered fiber-optic sensors with the same parameters, and the actual measurement results show that there were differences in the waist diameters of the five sensors within a standard deviation of less than 0.5 μm (see the Supplementary Material for the reproducibility of sensor fabrication). Overall, the tapered-in-tapered fiber-optic sensors prepared in the experiment had good repeatability.

### 2.4. Measurement System

Based on the design of a tapered-in-tapered fiber-optic sensor, an online transmission-type fiber-optic sensing system was constructed to facilitate the measurement of biological samples. Figure 2c shows the schematic diagram of the experimental setup for the proposed fiber biosensor. The broadband near-infrared light signal from the source (BBS, model: WBB400008SFA, Fiberlake Co., Ltd., Hongkong) was coupled into the tapered-in-tapered fiber-optic sensor, and after modulation by the measured sample, the sensing signal was transmitted to the spectrometer (OSA, model:AQ6370D, Yokogawa, Tokyo, Japan) for data acquisition. To ensure the stability of the sensor during the target molecule detection process, the sensor was fixed in a grooved glass slide using UV-curable adhesive. During the experimental detection process, a pipette was used to place the sample under test to avoid vibrations affecting the sensor during measurement, which could impact the results. For each measurement, the sample solution was first added to the sensing area using a pipette, ensuring that the sample solution was completely immersed in the sensing area, followed by spectral data collection. After measurement, the sensor was cleaned with a cleaning solution to ensure the accuracy of subsequent experimental results. Finally, the collected spectral data were processed and analyzed by a computer to obtain the detection results.

### 2.5. Materials

The materials used in this study were as follows: Single-mode fiber (SMF-28) was purchased from Corning (New York, NY, USA). Concentrated sulfuric acid solution (98%) and hydrogen peroxide solution (30%) were purchased from Dongguan Dongjiang Chemical Reagent Co., Ltd. (Dongguan, China). Ethanol (99.7%), glutaraldehyde, 3-aminopropyltriethoxysilane (APTES), phosphate-buffered saline (PBS, pH = 7.4), bovine serum albumin (BSA), ovalbumin (OVA), and casein were purchased from Shanghai Macklin Biochemical Technology Co., Ltd. (Shanghai, China), Sigma-Aldrich Company (St. Louis, MO, USA), and Sangon Biotech Co., Ltd. (Shanghai, China), respectively. Purified human IgG (H-IgG) was obtained from Origene Company (Rockville, MD, USA). Anti-h CRP and CRP were sourced from Medix Biochemica (Shanghai, China). The piranha solution was prepared by mixing concentrated sulfuric acid solution (98%) with hydrogen peroxide solution (30%) in a volume ratio of 3:1. All chemicals utilized in this research work were of analytical grade and were used without further purification.

## 3. Results and Discussion

### 3.1. Spectral Analysis

**Figure 3:**
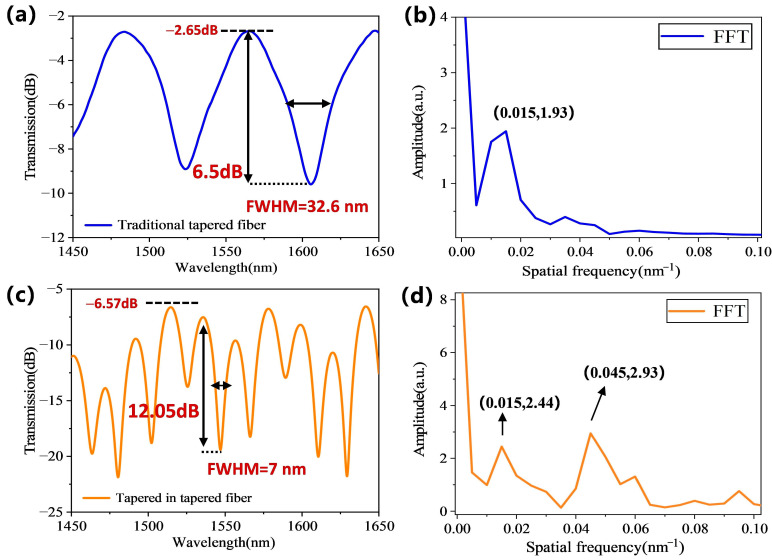
(**a**) Transmission spectra and (**b**) spatial frequency distribution of the traditional tapered fiber-optic sensor in deionized water. (**c**) Transmission spectra and (**d**) spatial frequency distribution of the tapered-in-tapered fiber-optic sensor in deionized water.

To further study the advantages of the tapered-in-tapered fiber-optic sensor, this part comparatively analyzed the spectral differences between traditional tapered fiber and tapered-in-tapered fiber before and after incorporating the built-in micro-tapered fiber structure in deionized water. Figure 3a presents the response transmission spectrum of the traditional tapered fiber-optic sensor in the wavelength range of 1450–1650 nm. It can be seen that the insertion loss of the traditional tapered fiber sensor was −2.65 dB, the interference fringe contrast was 6.5 dB, and the corresponding FWHM of the resonance peak was 32.6 nm. For a more intuitive analysis of spectral characteristics, Figure 3b shows the spatial frequency distribution of the spectrum obtained by fast Fourier transform (FFT). As a signal analysis method, in our experiment, the transmission spectrum data were converted into the spatial frequency domain to more intuitively analyze the interference signal of the sensor. The FFT analysis indicated that the interference signal of the traditional tapered fiber was mainly concentrated around 0.015 nm^−1^, exhibiting a typical two-beam interference pattern.

As for the tapered-in-tapered fiber-optic sensor, Figure 3c displays its detection response transmission spectrum. The results show a significant improvement in interference fringe contrast compared to the traditional tapered fiber-optic sensor, reaching 12.05 dB, with the corresponding FWHM narrowing down to 7 nm. The enhancement in interference fringe contrast can be attributed to the design of incorporating micro-tapered fiber within the tapered-in-tapered fiber structure, which not only strengthens the mode coupling effect of the fiber but also excites additional higher-order cladding modes. A narrower FWHM implies that the tapered-in-tapered fiber-optic sensor can respond more precisely to minor signal variations in wavelength-modulated target measurements [29]. The improvements in interference fringe contrast and FWHM performance significantly enhanced the detection accuracy and signal-to-noise ratio of the tapered-in-tapered fiber-optic sensor, thereby boosting the sensor’s resolution capability and stability. However, the insertion loss of the tapered-in-tapered fiber significantly increased to about −6.57 dB. The further increase in insertion loss can be attributed to light leakage due to the further reduction in the waist diameter, as well as additional transmission loss caused by the re-excitation of higher-order cladding modes.

Figure 3d illustrates the corresponding spatial frequency distribution of the tapered-in-tapered fiber-optic sensor. From the figure, it can be observed that the mode interference signals of the tapered-in-tapered fiber were concentrated at two frequencies: 0.015 nm^−1^ and 0.045 nm^−1^, indicating that the built-in micro-tapered fiber indeed introduced additional interference coupling modes. Moreover, compared to the traditional tapered fiber, the tapered-in-tapered fiber had an increased peak intensity at 0.015 nm^−1^. The built-in micro-tapered fiber not only enhanced the strength of low-frequency modes but also promoted the excitation of higher-order modes, thereby effectively improving the sensor’s responsiveness to subtle changes.

### 3.2. Sensor Performance

**Figure 4:**
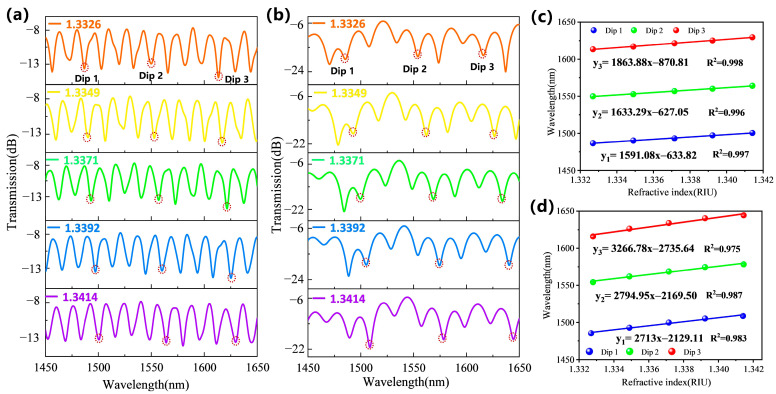
(**a**) Experimental detection spectra of the traditional tapered fiber-optic sensor (fiber waist diameter 7.25 µm) and (**b**) tapered-in-tapered fiber-optic sensor (micro-waist diameter 7.18 µm) under different RI environments. (**c**) RI sensitivity of the traditional tapered fiber-optic sensor and (**d**) tapered-in-tapered fiber-optic sensor.

To investigate the superiority of tapered-in-tapered fiber-optic sensors in detection sensitivity, we compared and analyzed the spectral response characteristics of the traditional tapered fiber-optic sensor and tapered-in-tapered fiber-optic sensor with the same minimum waist diameter under different liquid RI variations. A second traditional tapered fiber was fabricated with a waist diameter similar to that of the tapered-in-tapered fiber. The experimentally fabricated traditional tapered fiber had a waist diameter of 7.25 µm and a length of 20 mm, while the tapered-in-tapered fiber had a waist diameter of 7.18 µm and a length of 13 mm. Tapered-in-tapered fiber allows for a further reduction of the waist diameter while effectively preventing an increase in the overall sensor size. This optimized design reduced the amount of biological sample required for the assay. NaCl solutions with RIs of 1.3326, 1.3349, 1.3371, 1.3392, and 1.3414 were prepared and calibrated using an Abbe refractometer (INESA Instrument Physics & Optics Co., Ltd., Shanghai, China, WAY-2S) at a room temperature of 25 °C. Figure 4a,b show the spectral shifts for the traditional tapered fiber-optic sensor and the tapered-in-tapered fiber-optic sensor under different RIs, respectively. It can be observed that as the detected RI increased, redshifts occurred in the transmission spectra due to changes in the effective RI difference caused by external RI changes. For the traditional tapered fiber-optic sensor, the responses of Dip1, Dip2, and Dip3 with varying RI in the wavelength range of 1450–1650 nm were monitored, and the linear fitting analysis results are shown in Figure 4c. The RI sensitivities were found to be 1591.08 nm/RIU, 1633.29 nm/RIU, and 1863.88 nm/RIU, respectively. Dip3 exhibited the highest sensitivity, which was consistent with the higher sensitivity at longer wavelengths as described in Equation (3). The RI sensitivities for the tapered-in-tapered fiber-optic sensor are shown in Figure 4d, where the RI responses for Dip1, Dip2, and Dip3 were found to be 2713 nm/RIU, 2794.95 nm/RIU, and 3266.78 nm/RIU, respectively. Clearly, under the same fiber waist diameter, the tapered-in-tapered fiber structure significantly improved the RI detection sensitivity by approximately 1.7 times compared to the traditional tapered fiber structure.

The comparative experimental results demonstrate that the tapered-in-tapered fiber-optic sensor significantly outperformed the traditional tapered fiber in terms of performance. These improvements are primarily attributed to the additional interference coupling modes brought about by a micro-tapered fiber in a tapered fiber. Moreover, comparing the traditional tapered fiber and tapered-in-tapered fiber with identical waist diameters, not only was the sensitivity of the sensor been significantly improved, but the length of the sensing fiber was also reduced, which substantially decreased the sample volume required for detection. MAPE (mean absolute percentage error) is a commonly used error assessment indicator, typically employed to measure the relative error between predicted values and actual values [30]. We analyzed the RI detection accuracy of our sensor, as shown in the Table 1. From this, the MAPE was found to be 0.026%.

### 3.3. Sensor Surface Treatment and Immunofuntionalization for CRP Detection

**Figure 5:**
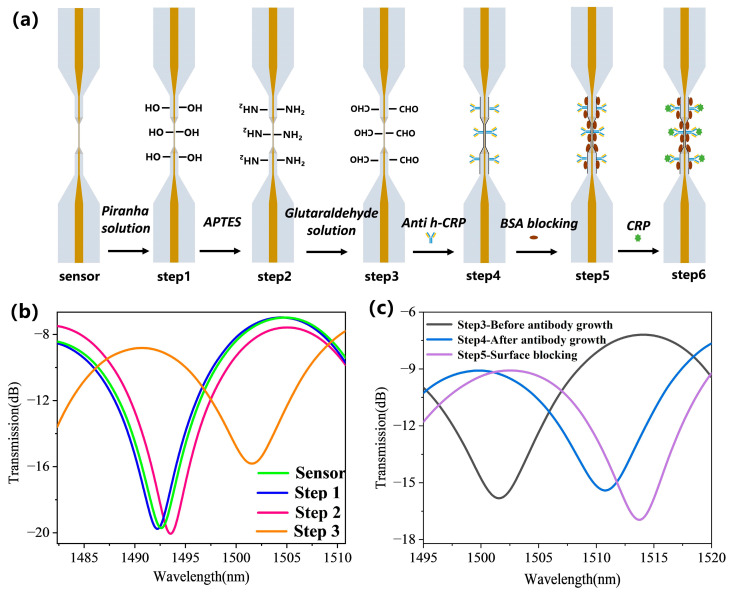
Bio-modification of the sensor: (**a**) Step-by-step process flow for sensor surface treatment and immunofuntionalization for CRP detection. (**b**) Sensing signal spectra during the surface treatment steps. (**c**) Sensing signal spectra during the immunofuntionalization steps.

In order to achieve specific detection of the target CRP by the proposed fiber-optic sensor, the modification of an immuno-recognition sensitive membrane on the sensor surface is particularly important. In this study, we employed glutaraldehyde crosslinking [31] to immobilize the CRP recognition molecule Anti-h CRP onto the sensor surface for immunofuntionalization. Figure 5a describes the specific fiber modification steps:

Step 1: The fiber-optic sensor, thoroughly cleaned with ethanol solution and deionized water, was immersed in piranha solution for 30 min, then rinsed several times with deionized water, and finally dried at room temperature. Piranha solution possesses strong oxidizing properties, effectively removing organic contaminants from the fiber surface and creating hydroxyl groups (-OH) on it.

Step 2: The fiber-optic sensor was immersed in a 10% (*v*/*v*) APTES ethanol solution for 2 h, then washed several times with ethanol solution, and finally dried at room temperature. The treatment with APTES ethanol solution introduces amino groups (-NH_3_) onto the fiber surface, providing chemical binding sites for subsequent coupling reactions.

Step 3: The fiber-optic sensor was immersed in a 10% (*v*/*v*) glutaraldehyde solution for 1 h, followed by several washes with PBS buffer. The purpose of the glutaraldehyde solution treatment is to further functionalize the fiber surface by introducing aldehyde groups (-CHO), enabling it to covalently bind with the amino groups of antibodies.

Step 4: The fiber-optic sensor was immersed in a 50 µg/mL Anti-h CRP solution for 1 h, anchoring antibody molecules to the fiber surface and endowing the fiber-optic sensor with the capability for specific CRP detection. Then, it was washed several times with PBS buffer to remove any unbound free antibody molecules.

Step 5: The fiber-optic sensor was immersed in a 1% (*w*/*v*) BSA solution for 30 min, followed by several washes with PBS buffer to remove any unbound protein molecules. The BSA solution treatment acts as a blocking step, preventing nonspecific reactions with other non-target molecules and enhancing the sensor’s resistance to interference.

Step 6: Once the surface functionalization is complete, the fiber-optic sensor can capture the target CRP, translating the antibody-antigen binding signal into a recognizable optical signal change.

Figure 5b,c records the interference signals after each step described above, showing that each step caused a significant redshift in the interference peaks. The surface modification process of protein molecules lead to a larger redshift.

### 3.4. CRP Detection Performance

**Figure 6:**
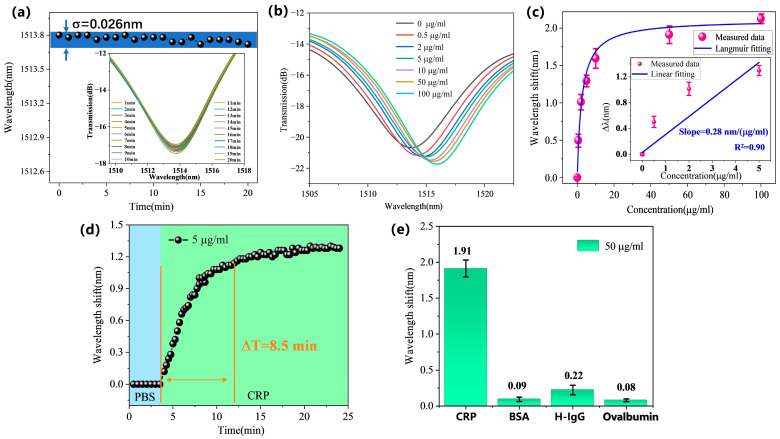
Biosensor detection results for target CRP: (**a**) Stability. (**b**) Sensing signal spectra at different concentrations of CRP. (**c**) Detection at different CRP concentrations. (**d**) Real-time detection of 5 µg/mL CRP. (**e**) Selectivity test, with all test sample concentrations at 50 µg/mL.

After the immunofunctionalization process, the stability of the fiber-optic biosensor was first assessed. The biosensor was immersed in PBS solution, and the response wavelength of the interference peak was monitored continuously for 20 min (collecting data every 1 min), as shown in Figure 6a. The results indicate that during the 20 min monitoring period, the standard deviation of the wavelength shift was 0.026 nm, demonstrating good stability of the sensor.

Next, to evaluate the sensitivity of the biosensor for CRP detection, CRP sample solutions with concentrations of 0.5 µg/mL, 2 µg/mL, 5 µg/mL, 10 µg/mL, 50 µg/mL, and 100 µg/mL were prepared using PBS as the background solution. These CRP solutions were injected onto the biosensor in ascending order of concentration, with a detection time of 30 min each. Notably, PBS buffer was used as the baseline reference before and after the detection of each concentration sample, and the sensor responses were recorded, as shown in Figure 6b. The detection results for different concentrations of CRP are shown in Figure 6c. As the detected CRP concentration increased, the sensor response wavelength change was consistent with the Langmuir adsorption curve model. In the low concentration range of 0–5 µg/mL, the sensor response was essentially linear, while in the high concentration range of 10–100 µg/mL, the sensor response tended toward saturation. After antibodies on the sensor surface captured CRP, the specific binding of CRP to Anti h-CRP caused an increase in the RI of the medium on the sensing area surface, resulting in a shift in the sensor response wavelength towards longer wavelengths. The inset in Figure 6c presents the results of a linear fitting analysis conducted within the low concentration range of 0–5 µg/mL for the CRP antigen. The analysis indicates a detection sensitivity of 0.28 nm/(µg/mL). The LOD is an important indicator of biosensor performance, aimed at evaluating the sensor’s ability to detect the minimum concentration of the target antigen. The calculated LOD for CRP antigen detection is 0.278 μg/mL by the formula of LOD=3σ/S [32],where σ = 0.026 nm is the standard deviation of the sensor signal under a blank sample and S is the linear slope of the sensor response signal versus target antigen concentration. To further verify the repeatability of the biosensor, three biosensors with the same parameters were prepared and subjected to CRP detection with the same gradient concentrations. The error bars in Figure 6c represent the standard deviation of wavelength response for the three sensors at the same CRP concentration, indicating good repeatability of our biosensor.

Response time and selectivity are also important parameters for evaluating biosensor performance [33]. To assess its response time, real-time monitoring of the wavelength response change was conducted during the detection of a 5 µg/mL CRP solution by our biosensor, as shown in Figure 6d. The results indicate that it took 8.5 min for the detection signal to reach 90% of its final stabilized state after the sensor captured CRP. Subsequently, the fluctuation amplitude did not exceed 1% over the following period. To further evaluate the selectivity of the biosensor, we tested its response to BSA, H-IgG, and OVA under the same experimental conditions. Figure 6e displays the response results of the biosensor to different protein molecules. It can be observed that the biosensor only had a significant response to CRP while showing minimal response to other protein molecules. This indicates that the tapered-in-tapered fiber-optic biosensor has good selectivity in CRP detection.

Finally, a comparison of different types of CRP biosensors is listed in Table 2. The long response time is mainly attributed to the efficiency of the antigen–antibody affinity binding reaction. To shorten the response time, on one hand, an oriented antibody immobilization method for sensor surface biomodification can be designed. Since the antibody immobilized by the biomodification method in this manuscript is not oriented, the binding sites of the antibody may face different directions, rather than uniformly and directionally exposing the antigen binding sites in the most favorable direction for antigen binding, resulting in hindered antigen–antibody binding. Oriented immobilization of antibodies can shorten the response time. On the other hand, antibodies with higher affinity can be sought to improve detection efficiency.

## 4. Conclusions

In this study, we introduce a Mach–Zehnder interferometer-based tapered-in-tapered fiber-optic biosensor. By integrating a micro-tapered fiber into a single tapered fiber structure, our design significantly enhanced sensitivity, signal-to-noise ratio, and resolution while reducing the length of the sensing fiber. The simulation analysis indicates that the tapered-in-tapered fiber markedly improved RI detection sensitivity by generating a stronger evanescent field effect. The experimental comparison between the tapered-in-tapered fiber and traditional tapered fiber revealed a 1.7-fold increase in sensitivity, achieving 3266.78 nm/RIU within the RI range of 1.3326 to 1.3414. Moreover, to expand its application potential in the biomedical field, we employed glutaraldehyde cross-linking technology to immobilize CRP antibodies on the surface of the tapered-in-tapered fiber, successfully creating a biosensing platform for the specific recognition of CRP. The experimental results demonstrate that this novel biosensor can rapidly and accurately detect CRP molecules at various concentrations, with a LOD of 0.278 μg/mL. Additionally, it exhibited excellent selectivity and reproducibility. This tapered-in-tapered fiber-optic biosensor provided valuable insights into the development of high-performance fiber-optic immunosensors and showed significant promise for applications in immunology research and early disease diagnosis.

## Figures and Tables

**Table 1:** Detection accuracy analysis of sensor.

Detection Number	1	2	3	4	5
Actual value (RIU)	1.3326	1.3349	1.3371	1.3392	1.3414
Measured value (RIU)	1.33224	1.33513	1.33753	1.33956	1.3410
Relative error	0.027%	0.017%	0.032%	0.027%	0.03%

**Table 2:** Performance comparison of CRP biosensors.

Sensor Type	Sensitivity	LOD	Detection Range	Response Time	Expense	Reference
Aptamer-based colorimetric assay	N.A. (Not applicable)	1.2 μg/mL	0.889–20.7 μg/mL	5 min	moderate	[34]
Fiber-optic SPR	2427.68 nm/RIU	0.22 μg/mL	0–78.6 μg/mL	15 min	expensive	[19]
Magnetic nanoparticles and capillary zone electrophoresis	N.A.	9.2 μg/mL	10–150 μg/mL	10 min	expensive	[35]
Fiber-optic LSPR	N.A.	0.024 ng/mL	0–2.5 μg/mL	N.A.	expensive	[36]
Tapered-in-tapered fiber	3266.78 nm/RIU	0.278 μg/mL	0–100 μg/mL	8.5 min	affordable	This work

## Data Availability

Data are contained within the article and Supplementary Materials.

## Supplementary Materials

The following supporting information can be downloaded at: https://www.mdpi.com/article/10.3390/bios15020090/s1. Figure S1: Simulated transmission spectra of tapered-in-tapered fibers with different waist diameters; Figure S2: Simulated RI sensitivity of tapered-in-tapered fibers with different waist diameters; Figure S3: The dimension scanning data of the five sensors fabricated in the experiment; Table S1: The tapered fiber waist diameters of the five sensors fabricated in the experiment.
