# Supplementary material for: Enhanced Sensitivity Mach–Zehnder Interferometer-Based Tapered-in-Tapered Fiber-Optic Biosensor for the Immunoassay of C-Reactive Protein
Source: Biosensors (Basel). 2025 Feb 6;15(2):90. doi: 10.3390/bios15020090 (PMC11853398; doi:10.3390/bios15020090)
Supplement: Supplementary file 1 [file biosensors-15-00090-s001.zip › biosensors-3420087-supplementary.pdf]

Supporting Information:

Enhanced Sensitivity Mach–Zehnder Interferometer-Based Tapered-in-Tapered Fiber-Optic Biosensor for the Immunoassay of C-Reactive Protein

Lei Xiao [1,2,3,†], Xinghong Chen [1,2,4,†], Xuejin Li [1,2,4,5], Jinghan Zhang [1,2,4], Yan Wang [1,2,4], Dongqing Li [1,2,4], Xueming Hong [1,2,4], Yonghong Shao [1,*] and Yuzhi Chen [1,2,4,*]

[1] College of Physics and Optoelectronic Engineering, Shenzhen University, Shenzhen 518060, China; 2050453015@email.szu.edu.cn (L.X.); 2250453009@email.szu.edu.cn (X.C.); lixuejin@szu.edu.cn (X.L.); zhangjinghan@mail.scuec.edu.cn (J.Z.); 2150453019@email.szu.edu.cn (Y.W.); 2210452034@email.szu.edu.cn (D.L.); xmhong@szu.edu.cn (X.H.)
[2] Shenzhen Engineering Laboratory for Optical Fiber Sensors and Networks, Shenzhen 518060, China
[3] Shenzhen Tian'an Zhiyuan Sensor Technology Co., Ltd., Shenzhen 518060, China
[4] Shenzhen Key Laboratory of Sensor Technology, Shenzhen 518060, China
[5] School of Science, The Chinese University of Hong Kong, Shenzhen 518172, China
* Correspondence: shaoyh@szu.edu.cn (Y.S.); chenyuzhi@szu.edu.cn (Y.C.)
† These authors contributed equally to this work.

1. Optimization Analysis of the Waist Diameter of the Micro-Tapered Fiber

To further enhance the rationality of the design, we conducted a simulation analysis on the response capability of different waist diameters of micro-tapered fiber sensors to external refractive index (RI) changes. Figure S1 (a) shows the response of a traditional tapered fiber (waist diameter: 20 μm) to different RIs, while Figure S1 (b-d) show the responses of micro-tapered fibers with different waist diameters (15 μm, 11 μm, and 7 μm) to different RIs. Figure S2 displays the RI sensitivity of micro-tapered fibers with different waist diameters.

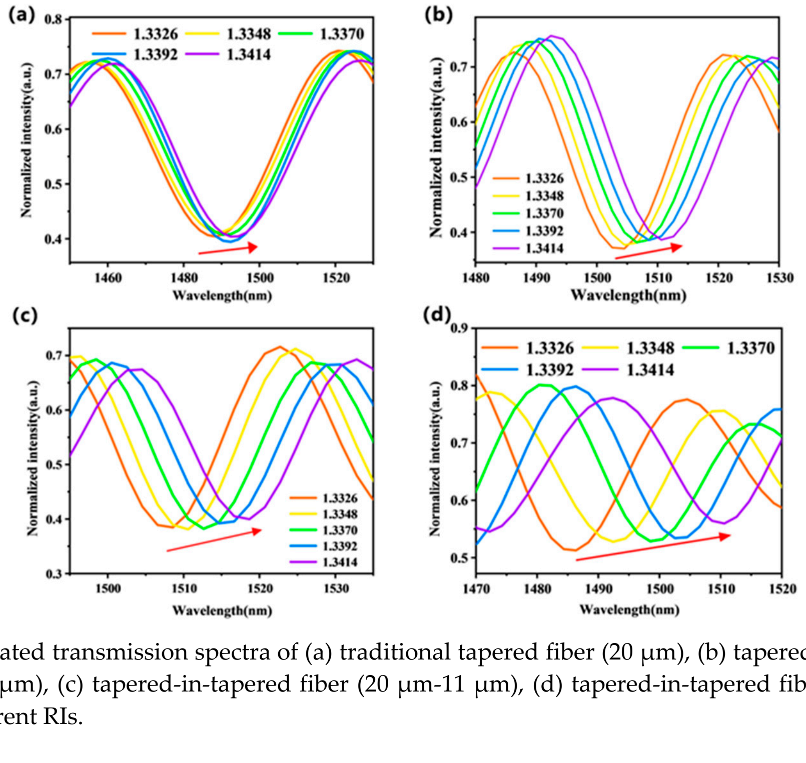

Figure S1. Simulated transmission spectra of (a) traditional tapered fiber (20 μm), (b) tapered-in-tapered fiber (20 μm-15 μm), (c) tapered-in-tapered fiber (20 μm-11 μm), (d) tapered-in-tapered fiber (20 μm-7 μm) under different RIs.

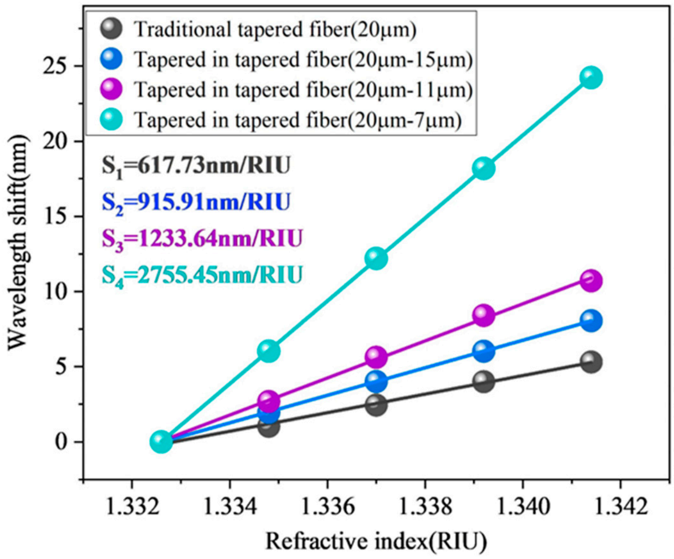

Figure S2. Simulated RI sensitivity of tapered-in-tapered fibers with different waist diameters.

2. Reproducibility of Sensor Fabrication

Five tapered-in-tapered fiber-optic sensors with the same parameters were experimentally prepared, and the actual measurement results show that there are differences in the waist diameters of the five sensors within a standard deviation of less than 0.5 μm, as shown in Figure S3 and Table S1.

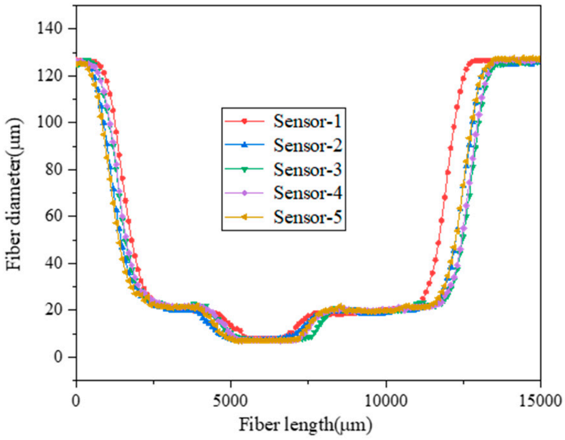

Figure S3. The dimension scanning data of the five sensors fabricated in the experiment.

Table S1. The tapered fiber waist diameters of the five sensors fabricated in the experiment.

Sensor number	1	2	3	4	5
Outer tapered fiber diameter (μm)	20.57	19.95	21.27	20.53	20.9
Inner tapered fiber diameter (μm)	8.04	7.42	7.64	7.19	7.07
